# TXN inhibitor impedes radioresistance of colorectal cancer cells with decreased ALDH1L2 expression via TXN/NF-κB signaling pathway

**DOI:** 10.1038/s41416-022-01835-1

**Published:** 2022-05-21

**Authors:** Lu Yu, Qiqing Guo, Ziqian Luo, Yongjia Wang, Jiawen Weng, Yuchuan Chen, Weijie Liang, Yiyi Li, Yuqin Zhang, Keli Chen, Zhenhui Chen, Yi Ding, Yaowei Zhang

**Affiliations:** 1 Department of Radiation Oncology, Nanfang Hospital, Southern Medical University, Guangzhou, China; 2 Huiqiao Medical Care, Nanfang Hospital, Southern Medical University, Guangzhou, China; 3 Department of Microbiology, Guangdong Provincial Key Laboratory of Tropical Disease Research, School of Public Health, Southern Medical University, Guangzhou, China

**Keywords:** Colorectal cancer, Radiotherapy

## Abstract

**Background:**

Colorectal cancer (CRC) is prevalent worldwide and is often challenged by treatment failure and recurrence due to resistance to radiotherapy. Here, we aimed to identify the elusive underlying molecular mechanisms of radioresistance in CRC.

**Methods:**

Weighted gene co-expression network analysis was used to identify potential radiation-related genes. Colony formation and comet assays and multi-target single-hit survival and xenograft animal models were used to validate the results obtained from the bioinformatic analysis. Immunohistochemistry was performed to examine the clinical characteristics of ALDH1L2. Co-immunoprecipitation, immunofluorescence and flow cytometry were used to understand the molecular mechanisms underlying radioresistance.

**Results:**

Bioinformatic analysis, in vitro, and in vivo experiments revealed that ALDH1L2 is a radiation-related gene, and a decrease in its expression induces radioresistance in CRC cells by inhibiting ROS-mediated apoptosis. Patients with low ALDH1L2 expression exhibit resistance to radiotherapy. Mechanistically, ALDH1L2 interacts with thioredoxin (TXN) and regulates the downstream NF-κB signaling pathway. PX-12, the TXN inhibitor, overcomes radioresistance due to decreased ALDH1L2.

**Conclusions:**

Our results provide valuable insights into the potential role of ALDH1L2 in CRC radiotherapy. We propose that the simultaneous application of TXN inhibitors and radiotherapy would significantly ameliorate the clinical outcomes of patients with CRC having low ALDH1L2.

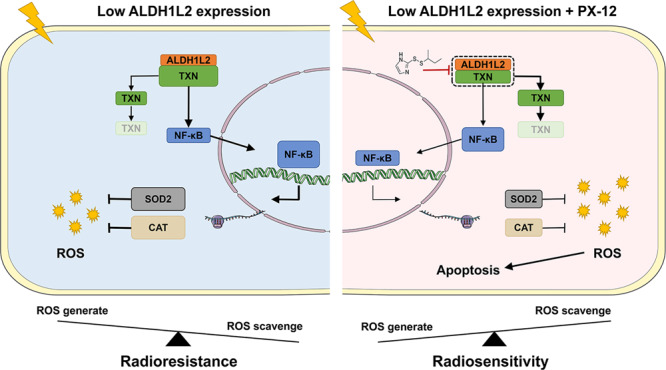

## Background

Colorectal cancer (CRC) is one of the most frequent malignant tumours worldwide, with the third and second highest incidence and mortality rates, respectively [1]. Currently, radiotherapy, as recommended by several cancer diagnoses and treatment guidelines, is an important treatment option for CRC [2–4]. Neoadjuvant radiotherapy combined with chemotherapy is the standard treatment for locally advanced rectal cancer and results in primary tumour size reduction, tumour downgrading and improvements in local tumour control rate in approximately two-thirds of treated patients [3–6]. In colon cancer, radiotherapy can be applied in the initial treatment of non-resectable, postoperative residual, or recurrent tumours, with beneficial effects [2, 7, 8]. However, radioresistance in tumour cells often causes radiotherapeutic failure, thereby impeding the achievement of the desired therapeutic outcome. Therefore, there is an urgent need to evaluate the molecular mechanisms underlying colorectal cancer radioresistance or radiosensitivity to improve patient prognosis.

Aldehyde dehydrogenase 1 family member L1 (ALDH1L1) is an important enzyme involved in folate metabolism, a candidate tumour suppressor and a potential marker for aggressive cancer [9, 10]. ALDH1 family member L2 (ALDH1L2) is a mitochondrial homolog of ALDH1L1, and the product of a separate gene on chromosome 12q23.3 [11]. ALDH1L1 and ALDH1L2 have similar structures and functions; they can catalyse 10-fTHF hydrolase and 10-fTHF dehydrogenase reactions [11–13]. However, being subject to compartmentalisation and having an additional unique sequence at its amino terminus, ALDH1L2 is involved in producing formate and regulating mitochondrial protein biosynthesis, NADPH production and oxidative stress [12]. Nevertheless, few studies have been conducted on the *ALDH1L2* gene, thus there is little information regarding it. It has been reported that ALDH1L2 provides tetrahydrofuran for the conversion of serine to glycine and for glycine degradation and that it participates in CoA-dependent pathways [14–16]. A previous study also found that ALDH1L2 is abnormally expressed in human colorectal and pancreatic cancer tissues and that this is associated with poor recurrence-free and overall survival in patients [15, 17]. Therefore, it is speculated that ALDH1L2 might play a crucial role in CRC occurrence and development. In addition, decreased ALDH1L2 expression has been reported in ALDH1L2 oxaliplatin-resistant CRC and ovarian cancer cell lines [18]. We also previously demonstrated that reduction in ALDH1L2 expression substantially affects the phenotype of radioresistance in HCT 116 and SW480 CRC cell lines in vitro [19], but there was no detailed validation of the underlying mechanism. Moreover, the intricate correlation between decreased ALDH1L2 and induction of radioresistance in CRC cell lines is not yet explored. Therefore, our aim was to identify these underlying mechanisms.

TXN is a classic redox protein that can regulate the cell redox state in several ways; TXN can eliminate reactive oxygen species (ROS) to protect cells from oxidative stress [20, 21]. In addition, TXN can interact with proteins containing cysteine (Cys) residues to modify their conformation and function [21, 22]. In response to nitric oxide, TXN nitrosylates the Cys active site of CASP3, thereby inhibiting its activity and, consequently, cell apoptosis [23]. Previous studies have shown that TXN directly interacts with the p50/p65 heterodimer in the nucleus and regulates NF-κB activity [24–26]. In this study, we also tried to explore whether TXN contributes to the development of radioresistance in CRC cell lines caused by the reduced expression of ALDH1L2.

In this study, we evaluated irradiation-related genes using bioinformatic methods and predicted that ALDH1L2, along with 3 other genes, is a radiosensitivity-related gene. Through in vitro and in vivo experiments, and clinical tissue analysis, we defined ALDH1L2 as a radiosensitive gene by describing the TXN/NF-κB pathway-mediated CRC cell apoptosis. These findings suggest that in CRC patients with low ALDH1L2 expression levels, who may be resistant to radiotherapy, the simultaneous application of TXN inhibitors and radiotherapy may be an effective treatment strategy; this may help improve radiosensitivity, allowing patients to benefit more from treatment.

## Materials and methods

### Reagents and materials

The reagent suppliers are indicated in Table S1.

### Bioinformatic analysis

Raw messenger RNA (mRNA) expression profiles and clinical features of the GSE46862, GSE36133, GSE14333, GSE93375, GSE133057 and TCGA datasets were downloaded from the GEO database (http://www.ncbi.nlm.nih.gov/geo/) and National Cancer Institute website (https://portal.gdc.cancer.gov/). Weighted gene co-expression network analysis (WGCNA) was used to generate a co-occurrence network based on RNA-seq robust multiarray averaging background correction data [27]. A total of 3000 genes (ranked by variation) in the GSE46862 and GSE36133 datasets were screened. Gene set enrichment analysis (GSEA) was performed on the TCGA dataset using a software package downloaded from www.broadinstitute.org/gsea [28]. The number of permutations was set to 1000. Protein–protein docking was conducted using the online “HDCOK server” (http://hdock.phys.hust.edu.cn/) [29]. The correlation between genes and infiltrating immune cells in the TCGA dataset was determined using the online GEPIA platform (http://gepia.cancer-pku.cn/) [30].

### RNA isolation and qRT-PCR

Total RNA was extracted using the TRIzol reagent (TaKaRa Bio, Tokyo, Japan) following the manufacturer’s instructions. cDNA was generated using a one-step reverse transcriptase cDNA synthesis kit. mRNA expression was analysed using a SYBR Green PCR Kit on the 7500 Fast Real-time PCR system (Applied Biosystems, Thermo Fisher Scientific, Waltham, MA, USA) and β-actin was used for normalisation. Data were analysed using the ^△△^CT method. The primers used for PCR are shown in Table S2.

### Protein extraction and western blotting

Proteins were extracted from cells using a protein extraction kit or a nuclear and cytoplasmic protein extraction kit and subsequently used for western blot analysis. Western blotting was performed following the manufacturer’s instructions.

### Cell culture, lentivirus infection and siRNA transfection

CRC cell lines SW480, HT-29, HCT 116, RKO, SW620, Caco-2 and HCT-15 were purchased from ATCC (Manassas, VA, USA). All cell lines were cultured in RPMI-1640 medium (GIBCO BRL, Grand Island, NY, USA) containing 10% FBS (GIBCO BRL). Cells were cultured at 37 °C with 5% CO_2_. Lentiviral full-length expression ALDH1L2 and ALDH1L2 targeting short hairpin RNAs (CTGTGTTCAAGCTTCCTAAATGG) with GFP and FLAG were all constructed by VectorBuilder (Guangzhou, China). ALDH1L1 targeting siRNAs (#1: GTGCCATAAGTAACGTGAA, #2: GGGCAAGCACATCATGA-AA, #3: GGAGGACTCCATTCATGAT) were all constructed by RiboBio (Guangzhou, China). Infection and in vitro transfection were conducted on cell lines following the manufacturer’s protocol. Cells were treated with pyrrolidine dithiocarbamate ammonium (PDTC) or 1-methyl propyl 2-imidazolyl disulfide (PX-12) at the concentration of 100 or 20 μM, respectively, (Selleck Chemicals, Houston, TX, USA).

### Colony formation assay and the multi-target single-hit survival model

The radiosensitivity of cell lines was determined through a colony formation assay by applying the multi-target single-hit model to the surviving fractions [31, 32]. Cells were plated in six-well plates and irradiated at doses of 0, 2, 4, 6 and 8 Gy (6 MeV X-ray, Varian, PaloAlto, CA, USA). Then, the cells were cultured for 14 days, after which the colonies were stained with 0.5% crystal violet and quantified using the ImageJ software version 1.8.0 (Bethesda, MD, USA). The surviving fraction at each dose was calculated using the formula:

[(number of surviving colonies in dose X) / (number of cells seeded for dose X (average colonies arising from the non-irradiated cells (0 Gy)) / number of non-irradiated cells seeded)].

Survival curves were used to develop the multi-target single-hit model, SF = 1 − (1 − e^−D/D0^) × N, where SF is the surviving fraction and D is the radiation dose and N is the extrapolation number [31].

### Comet assay

The comet assay was performed in accordance with the manufacturer’s protocol. Briefly, slides were covered with 200 μL of pre-warmed normal melting point agarose (2%) and placed on ice for the solidification of the first gel layer. Then, cells irradiated at 6 Gy were digested to obtain a cell suspension. Ten microliters of the cell suspension were mixed with 190 μL of pre-warmed low melting point agarose (0.75%) and poured onto the slides. Next, the slides were dipped in a cold lysis solution for 1 h. After cell lysis, the slides were placed in a horizontal electrophoresis chamber (Bio-Rad Laboratories, Hercules, CA, USA) filled with cold TAE solution and incubated for 20 min in the dark and then electrophoresed (1 V/cm, 25 min). Then, the slides were neutralised in PBS for 5 min, stained with propidium iodide (PI), and the comet images were captured using a fluorescence microscope (Olympus Corporation, Tokyo, Japan). The percentage of DNA in the tail was analysed using the CASP 1.2.3 beta 1 software (Krzysztof Konca, Poland) [33].

### Animal model

The animal studies were approved by the Institutional Animal Care and Use Committee of Nanfang Hospital. Male BALB/c nude mice (4 weeks old, *n* = 71) were purchased from Southern Medical University Laboratory Animal Center, China, and raised under specific pathogen-free conditions. All in vivo experiments were performed in accordance with our institution’s guidelines on the use of laboratory animals. The mice were randomly divided into groups with no blinding. To develop the xenograft tumour model, 5 × 10^6^ cells were subcutaneously injected into the left flanks of the mice. When tumours reached a volume of 100 mm^3^, they were irradiated at 6 Gy once or twice. Only the tumours were irradiated, and the other parts of the mouse’s body were protected with a lead shield. A TXN inhibitor, PX-12 (12 mg/kg, Selleck Chemicals, Houston, TX, USA), was intraperitoneally injected into the mice daily and 3 h before irradiation. Tumour volume was measured using Vernier calipers and calculated as 1/2 × length × width × width. After the mice were euthanised using phenobarbital sodium, the tumours were excised, weighed and embedded in paraffin for further experiments.

### Immunohistochemistry

Through mouse model tumour immunohistochemistry (IHC) staining, ki-67 protein expression was determined using mouse anti-Ki-67 monoclonal antibodies (1:800). For patient tissue IHC staining, all rectal adenocarcinoma tissues collected before treatment, 4 μm sections and information on clinicopathological features of patients were provided by the Department of Pathology, Nanfang Hospital of Southern Medical University (*n* = 21). ALDH1L2 protein expression was determined using rabbit anti-ALDH1L2 polyclonal antibodies (1:150). Staining intensity was independently evaluated by two senior pathologists. Specimen collection was approved by the Ethics Committee of Nanfang Hospital, Southern Medical University, Guangdong Province, China.

### Flow cytometric analysis of ROS and apoptosis

ROS were stained with dihydroethidium (DHE) by flow cytometry (BD Biosciences, San Jose, CA, USA). In brief, the cells were irradiated at 6 Gy and incubated at 37 °C for 30 min in 1640 medium containing 1 μM DHE. After incubation with several fluorescent probes, the cells were washed twice with PBS and analysed by flow cytometry. The cell cycle was also analysed by flow cytometry; for this, cells were irradiated at 6 Gy, digested with trypsin in the absence of EDTA for 24 h, washed twice with PBS, incubated with Annexin-V APC probes and PI dye and finally analysed by flow cytometry. The percentage of apoptotic cells was calculated by adding the percentage of cells in Q2 and Q3 quadrants.

### Co-immunoprecipitation (IP) assay, LC-MS and silver staining

Total protein was extracted as previously described [34], and 100 μg of the lysate was incubated overnight with mouse anti-FLAG (1:200) monoclonal antibodies, rabbit anti-TXN (1:50) polyclonal antibodies, or IgG (as a negative control, 1:1000) at 4 °C on a rocker platform. Then, the resulting complex was incubated with 20 µL of Protein A/G PLUS-Agarose for 5 h at 4 °C. Complex beads were collected by centrifugation at 1000 × *g* for 5 min at 4 °C and washed four times with PBS. After the final wash, the supernatant was aspirated and discarded, and the remaining pellet was resuspended in 5× electrophoresis sample buffer. After the samples were boiled for 3 min, aliquots were made and analysed by SDS-PAGE.

After protein separation by SDS-PAGE, gels containing IP complexes were stained with Coomassie blue. The gel lane was divided into fractions and cut into small pieces (~1 mm^3^). Then, the Coomassie blue stain and SDS were washed off. The gel pieces were dehydrated and rehydrated with a trypsin solution for protein digestion, and the mix was incubated overnight. Finally, peptides were eluted from the gels and detected using an LS-MS Orbitrap Fusion Tribrid system (Thermo Fisher Scientific, Waltham, MA, USA).

Gel silver staining was performed according to the protocol described by Beyotime Technology. In brief, after electrophoresis, the gels were washed in 30% ethanol for 10 min and then in Milli-Q water for another 10 min. Then, the gels were incubated with a silver staining sensitiser for 2 min and then with silver nitrate for 10 min. Next, the gels were placed in Milli-Q water for 1 min, and then removed and placed in the developing solution. When clear staining was achieved, the gels were transferred to a stop solution and incubated for 10 min. Then, the stained gels were photographed.

### Immunofluorescence analysis

Immunofluorescence (IF) experiments were performed using rabbit anti-TXN polyclonal antibodies (1:100) and goat anti-rabbit IgG/Alexa Fluor 594 antibodies as primary and secondary antibodies, respectively, following the manufacturer’s protocol. Cell nuclei were counterstained with 200 µL of DAPI fluorescent dye. Fluorescent signals were observed using an Olympus FV3000 confocal microscope (Olympus Corporation, Tokyo, Japan).

### Statistical analysis

Data are expressed as the mean ± standard deviation (SD), and *P*-values <0.05 were considered statistically significant in all experiments. Data were analysed using one-way analysis of variance (ANOVA), Spearman’s correlation, and Kaplan–Meier estimations. Statistical analysis was performed using the SPSS 20.0 software (IBM, Armonk, NY, USA). All experiments were performed thrice.

## Results

### Bioinformatic evaluation of radiation-related genes in CRC cells

**Fig. 1:**
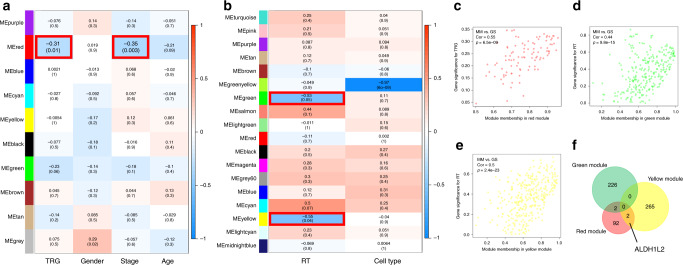
Identification of key modules correlated with irradiation in GSE46862 and GSE36133 through WGCNA. **a** Heatmap of the correlation between module eigengenes and clinical traits in GSE46862. **b** Heatmap of the correlation between module eigengenes and clinical traits in GSE36133. Each cell contains the correlation coefficient and *P*-value. Scatter plot of module eigengenes in the **c** red module, **d** green module and **e** yellow module. **f** Veen map of commonly shared genes in the above three modules.

Bioinformatics tools, especially WGCNA, allow the identification of genes associated with certain phenotypes among many. To identify genes related to radiation response, WGCNA was performed on the GSE46862 and GSE36133 datasets. The GSE46862 dataset was composed of 69 rectal cancer patients and their clinical data, including their TRG scores, as shown in Fig. S1A and Table S3. A threshold power (β) of 13 was systematically selected for the construction of a scale-free network (Fig. S1B–D). Aside from the grey module, nine other modules were identified (Fig. 1a). The GSE36133 dataset was composed of 14 CRC cell lines, and the response to radiation in these is shown in Fig. S1E and Table S4. β of 10 was selected (Fig. S1F–H), and a total of 18 modules were identified (Fig. 1b). Of these modules, the red module from GSE46862 was the only one significantly negatively correlated with the TRG score, indicating that these genes are negatively correlated with response to neoadjuvant chemoradiotherapy (r = −0.31; *P* = 0.01; *n* = 96 genes; Fig. 1a and Fig. S1I). Interestingly, this module was also associated with tumour stage (r = −0.35; *P* = 0.003; Fig. 1a). The green (r = −0.53; *P* = 0.05; *n* = 228 genes) and yellow (r = −0.55; *P* = 0.04; *n* = 267 genes) modules from GSE36133 were significantly negatively correlated with the response to radiation (Fig. 1b and S1J). The correlations between module membership (MM) and the gene significance (GS) values of the red, green and yellow modules, along with their respective P-values, are shown in Fig. 1c–e and S1K. Genes common to these three key modules in the GSE46862 and GSE36133 datasets are shown in Fig. 1f and S1L and are all negatively correlated with radiation. Thus, the bioinformatic analysis predicted that four genes, including *ALDH1L2*, are related to radiosensitivity in CRC cells.

### *ALDH1L2* plays a radiosensitive role in CRC cells

**Fig. 2:**
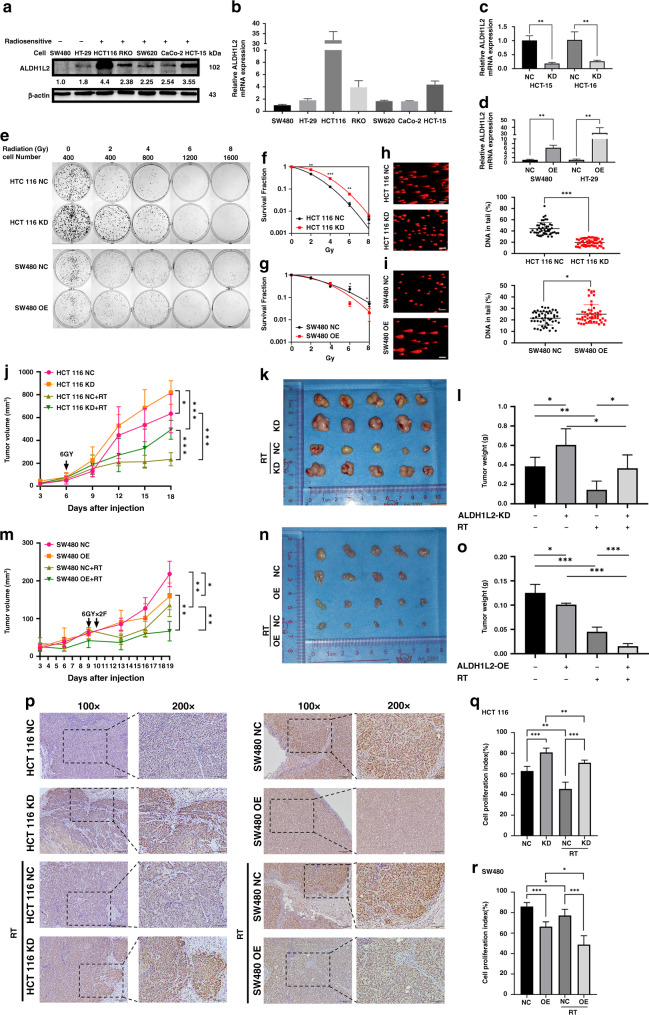
ALDH1L2 is a radiosensitive gene in vitro and in vivo. **a** Evaluation of protein expression by western blotting in seven colorectal cancer cell lines. β-actin served as a loading control. **b** Different ALDH1L2 transcript levels of seven CRC cells. **c** QRT-PCR for ALDH1L2 in HCT-15 and HCT 116 cell line after transfected with knockdown lentivirus. **d** QRT-PCR for ALDH1L2 in SW480 and HT-29 cell line after transfected with overexpression lentivirus. **e** Colony formation assay on different cell lines and its negative control. The survival curves of multi-target single-hit model on **f** HCT 116 cell lines and **g** SW480 cell lines. **h**, **i** Comet assay on different cell lines and its negative control. **j** Growth of tumour volume, **k** image and **l** weight of tumours harvested in nude mice injected with ALDH1L2-knockdown cell lines and its control, with/without 6 Gy irradiation. **m** Growth of tumour volume, **n** image and **o** weight of tumours harvested in nude mice injected with ALDH1L2 overexpressed cell lines and its control, with/without 12 Gy irradiation. **p** Immunofluorescence staining of Ki-67 in tumours from HCT 116 cell lines with ALDH1L2 knockdown and SW480 cell lines with ALDH1L2 overexpressed and their negative control, with irradiation or not. **q** Bar chart demonstrating the Ki-67 expression of HCT 116 cell lines. **r** Bar chart demonstrating the Ki-67 expression of the SW480 cell lines. *P < 0.05; **P < 0.01; ***P < 0.001.

Three of the four identified genes have been reported to be radiation-related; therefore, we focused on the last gene, *ALDH1L2*. We used two radioresistant CRC cell lines and five radiosensitive CRC cell lines to evaluate ALDH1L2 mRNA and protein levels. Western blotting and qRT-PCR showed that SW480 and HT-29 cells exhibited lower ALDH1L2 expression levels than other cells, while the HCT 116 and HCT-15 cell lines exhibited higher ALDH1L2 expression levels than the other CRC cell lines (Fig. 2a, b). To investigate the biological functions of ALDH1L2, SW480 and HT-29 cells were transfected with ALDH1L2-overexpressing lentiviruses. ALDH1L2 expression was suppressed in HCT 116 and HCT-15 cells through stable transfection with shALDH1L2 lentiviruses. ALDH1L2 expression levels were determined by qRT-PCR and western blotting (Fig. 2c, d and 4c). Using a colony formation assay and the multi-target single-hit survival model, we found that ALDH1L2 knockdown induced higher colony numbers and lower radiosensitivity (Fig. 2e, f and S2A, B). In addition, opposite results were observed following ALDH1L2 overexpression (Fig. 2e, g and S2C, D). Through the comet assay, we found that cells exhibited shorter tails and less DNA damage following ALDH1L2 knockdown (Fig. 2h and S2E). We also found that cells exhibited longer tails and more DNA damage following ALDH1L2 overexpression (Fig. 2I and S2F). Since ALDH1L2 is a homolog of ALDH1L1, we used siRNA, western blot, and qRT-PCR to explore whether there is any compensation between homologs after inhibition of each gene. The results indicated that the protein and mRNA levels of ALDH1L2 remained unchanged after ALDH1L1 was inhibited (Fig. S3A, B). Moreover, the protein level of ALDH1L1 was unchanged when cells were transfected with shALDH1L2 lentiviruses (Fig. 4c). Thus, ALDH1L2 was found to play a radiosensitive role in CRC cells in vitro independent of ALDH1L1.

To determine whether *ALDH1L2* inhibits radioresistance in vivo, we employed a xenograft colorectal cancer model. We found that the volumes and weights of the tumours harvested from mice in the “ALDH1L2 silencing” group were significantly higher than those of mice in the control group, regardless of irradiation (Fig. 2j–l). We obtained opposite results in mice in the “ALDH1L2 overexpressing” group (Fig. 2m–o). Then, these tumours were examined through IHC staining using anti-ki-67 antibodies. We observed that both the percentage of positively stained cells and the intensity of Ki-67 staining were significantly high in ALDH1L2-knockdown tumours, even after irradiation (Fig. 2p, left, q). The opposite was observed in ALDH1L2-overexpressing tumours, even after irradiation (Fig. 2p, right, r). These data support the speculation that ALDH1L2 inhibits radioresistance and proliferation in vivo.

### Decreased ALDH1L2 expression induces resistance to neoadjuvant radiochemotherapy in patients with rectal cancer

**Fig. 3:**
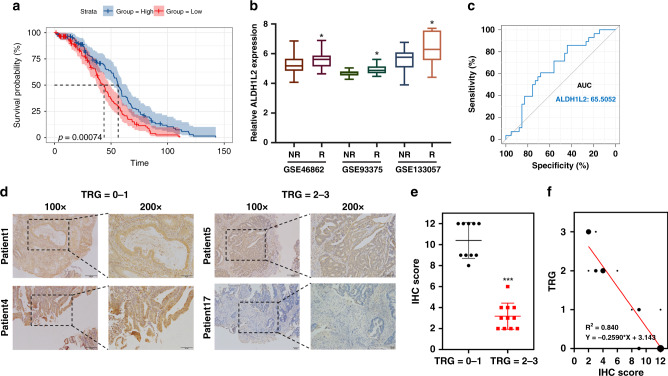
Rectal cancer patients with low ALDH1L2 expression are not responsive to neoadjuvant chemoradiotherapy. **a** Kaplan–Meier survival curve of GSE14333 (*n* = 290). **b** Gene expression was obtained and compared in 3 GEO datasets. **c** ROC curve of ALDH1L2 expression in GSE46862. **d** Representative IHC staining of ALDH1L2 in rectal cancer tissues with different TRG scores. **e** IHC scores in different TRG score groups. **f** Correlation between TRG scores and IHC scores. *P < 0.05; **P < 0.01; ***P < 0.001.

To further assess whether ALDH1L2 expression is associated with the clinical characteristics of CRC patients, Kaplan–Meier estimation and expression analyses by stage were conducted. The Kaplan–Meier plot derived from the GSE14333 dataset demonstrated that a lower ALDH1L2 expression is closely associated with shorter disease-free survival of CRC patients (Fig. 3a). We then assessed the expression of ALDH1L2 mRNA in the GEO datasets, GSE46862, GSE93375 and GSE133057, observing that ALDH1L2 mRNA levels were significantly lower in patients with rectal cancer who were non-responsive to neoadjuvant radiochemotherapy than in responsive patients (Fig. 3b). The receiver operating characteristic curve (ROC) was derived from the GSE46862 dataset, and the area under the curve (AUC) of ALDH1L2 for predicting pathological response was 0.655 (Fig. 3c). Consistent with the results obtained from analyzing the GEO data, ALDH1L2 protein levels, as determined by IHC staining, were downregulated in rectal cancer tumours with a TRG score of 2 or 3 after treatment with neoadjuvant chemoradiotherapy (Fig. 3d, e, and Table S5). In addition, ALDH1L2 expression was negatively correlated with the TRG score (R^2^ = 0.840; *P* < 0.0001; Fig. 3f).

### Knockdown of ALDH1L2 inhibits ROS-mediated apoptosis

**Fig. 4:**
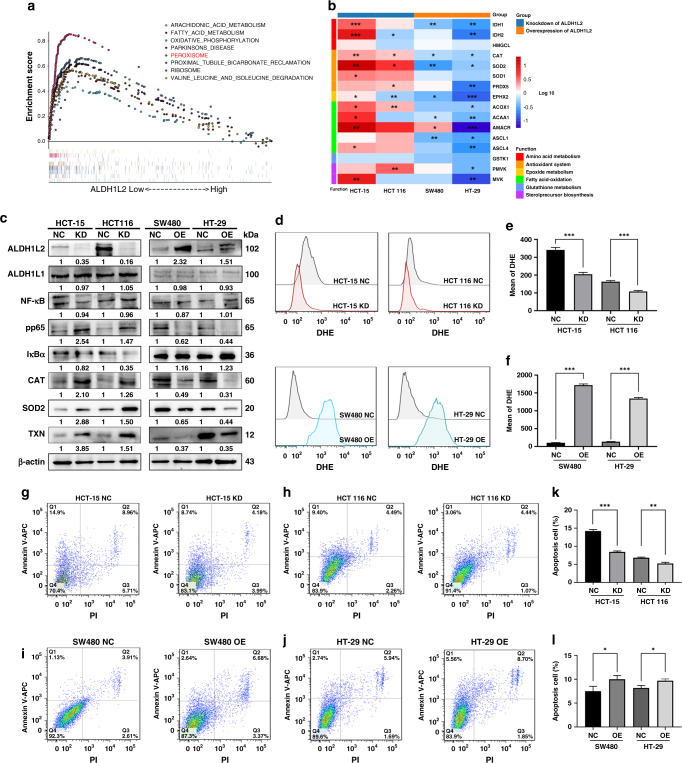
Decreased ALDH1L2 inhibits ROS-mediated apoptosis in CRC cells after irradiation. **a** GSEA enrichment plot of CRC samples from TCGA dataset. NES and *P*-value were calculated in the plot. **b** Heatmap showing the transcription level of genes in peroxisome pathway. Cells were treated with 6 Gy irradiation. *P*-value were shown in the plot. **c** Protein expression of ALDH1L2, NF-κB, pp65, iκBα, CAT, SOD2 and TXN with silencing ALDH1L2 in HCT-15 and HCT 116 cells or forced expression of ALDH1L2 in SW480 and HT-29 cell. β-actin served as a control. **d** Overlay of histograms by flow cytometry. Mean of DHE in **e** cells with ALDH1L2 knockdown or **f** cells with ALDH1L2 overexpressed. **g–j** Apoptosis of different cell lines and its negative control by flow cytometry. The percentage of cells in all quarters were addressed. Cells were treated with 6 Gy irradiation. **k**, **l** Bar graph to show the percentage of apoptosis cells after irradiation. *P < 0.05; **P < 0.01; ***P < 0.001.

To circumvent the problem of radioresistance, we conducted GSEA on the TCGA dataset to identify cellular pathways related to low ALDH1L2 expression. The identified enriched KEGG pathways are shown in Fig. 4a. Of all the KEGG pathways with a *P*-value <0.05, the peroxisome and fatty acid metabolism pathways were reported to be closely related to irradiation [35, 36]. After 6 Gy irradiation, we used qRT-PCR to determine the mRNA levels of genes involved in these two pathways in HCT-15, HCT 116, SW480 and HT-29 cell lines with ALDH1L2 silencing or overexpression (Fig. 4b and S4A–D). Upon irradiation, the mRNA levels of genes involved in the peroxisome pathway were changed, most of which were members of the antioxidant system (Fig. 4b). Western blotting indicated that the protein levels of the classic antioxidant enzymes, CAT and SOD2, were significantly upregulated following ALDH1L2 knockdown, and observations opposite to these were made in ALDH1L2 overexpression cells (Fig. 4c). To further investigate how *ALDH1L2* affects cell fate after irradiation, we used flow cytometry to evaluate the levels of ROS, which are non-negligible products found in cells following irradiation and apoptosis. Mean DHE levels decreased when *ALDH1L2* was inhibited and significantly increased when ALDH1L2 expression was promoted (Fig. 4d–f). In addition, flow cytometric analysis indicated that *ALDH1L2* promoted cell apoptosis after irradiation at 6 Gy (Fig. 4g–l). Of note, following the administration of NAC, a typical ROS scavenger, apoptosis was reversed in ALDH1L2-overexpressing cells (Fig. S4E–H). Collectively, these findings suggest that knockdown of ALDH1L2 inhibits ROS-mediated apoptosis.

### ALDH1L2 interacts with TXN and regulates protein degradation

**Fig. 5:**
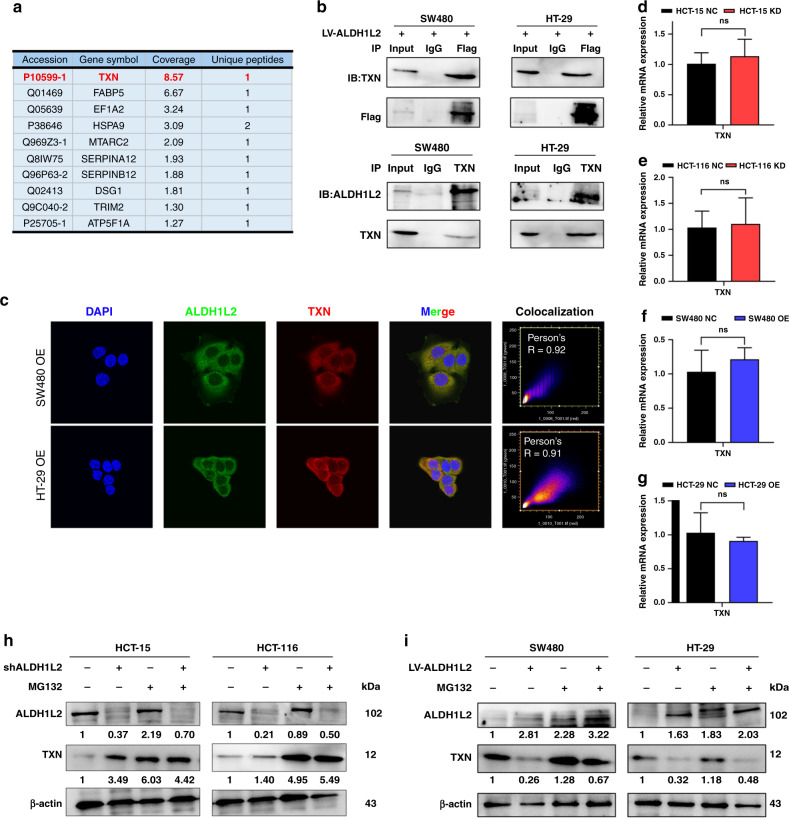
ALDH1L2 interacts with TXN. **a** Results of LC-MS. **b** Co-IP identified the interaction between ALDH1L2 and TXN. **c** IF analysis of the co-localisation of ALDH1L2 (green) and TXN (red) in SW480 and HT-29 cell lines with ALDH1L2 overexpression. Nucleus was labelled with DAPI (blue). **d–g** Transcript level of TXN in CRC cell lines with different ALDH1L2 expressions. **h** MG132 (20 μM, 24 h) treatment on HCT 116 and HCT-15 cells with/without ALDH1L2 knockdown. **i** MG132 (20 μM, 24 h) treatment on SW480 and HT-29 cells with/without ALDH1L2 overexpression. *P < 0.05; **P < 0.01; ***P < 0.001.

To elucidate the molecular mechanisms underlying the inhibitory effects of ALDH1L2 on the peroxisome pathway, we conducted Co-IP and gel silver staining analyses and prepared sections from the gels to determine peptide levels by LC-MS (Fig. 5a and S5A). We found that after ALDH1L2, TXN was the most abundant IP protein product (Fig. 5a). Western blotting was performed to compare SW480 and HT-29 cell lysate anti-FLAG IP with their anti-IgG IP products, and TXN was found to interact with ALDH1L2 (Fig. 5b, upper). To further verify this endogenous interaction, Co-IP was performed by incubating SW480 and HT-29 cell lysates with anti-TXN antibodies, and it was found that ALDH1L2 could be equally co-precipitated by TXN in both cell lines (Fig. 5b, lower). The potential docking site is shown in Fig. S5B. Next, we investigated the subcellular locations of ALDH1L2 and TXN by IF and found that both their signals overlapped in CRC cell cytoplasm, especially in the mitochondria (Fig. 5c). Based on this finding, we performed western blotting and qRT-PCR to determine TXN protein and mRNA levels in ALDH1L2-knockdown and ALDH1L2-overexpressing cell lines. TXN protein expression was found to be upregulated in ALDH1L2-knockdown cell lines and downregulated in the ALDH1L2 overexpressing (Fig. 4c). Interestingly, TXN mRNA levels were unchanged despite the modification of ALDH1L2 expression (Fig. 5d–g). It is well-known that this discrepancy between transcription and protein levels might be caused by protein degradation or changes occurring during translation. Thus, we pretreated cells with a proteasome inhibitor, MG132, and found that it induced a significant increase in TXN protein expression in control cells, with only a slight upregulation in TXN levels observed in ALDH1L2-knockdown cell lines (Fig. 5h–i). These results suggest that ALDH1L2 interacts with TXN and regulates its protein degradation.

### TXN inhibitor restricts the development of radioresistance in CRC cells with lower ALDH1L2 expression

**Fig. 6:**
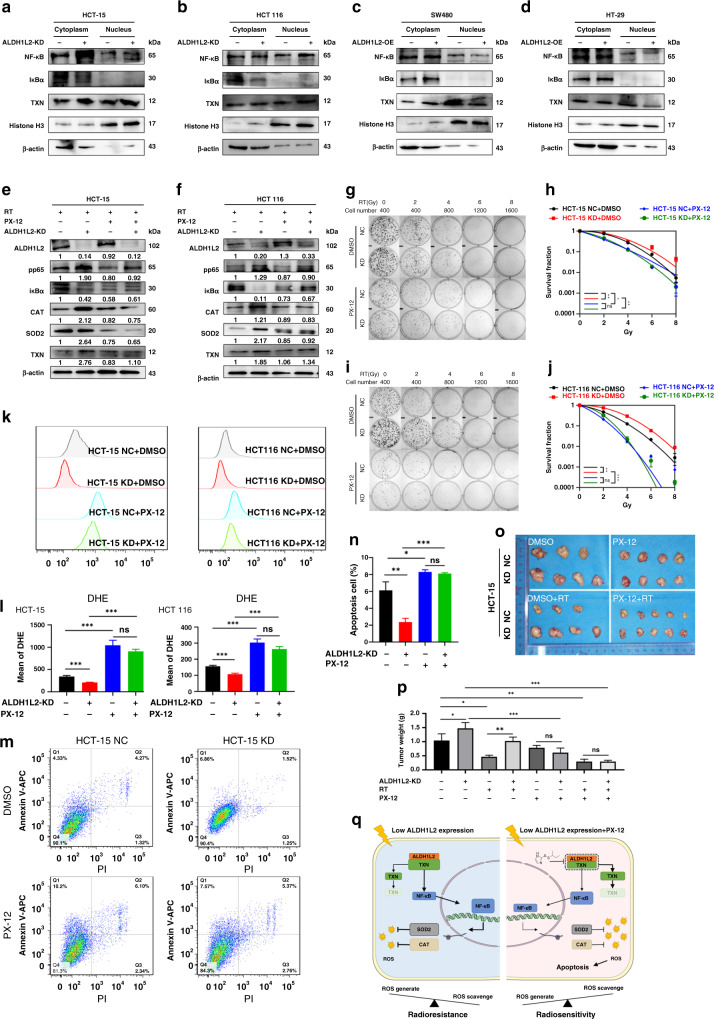
TXN inhibitor overcomes the decreased ALDH1L2 expression mediated radioresistance through promoting the downstream NF-κB signaling pathway. **a**, **b** Western blotting of indicated proteins with/ without ALDH1L2 knockdown after 6 Gy irradiation. **c**, **d**) Western blotting of indicated proteins with/ without ALDH1L2 overexpression after 6 Gy irradiation. Histone H3 was a nuclear marker and β-actin was served as a cytoplasmic marker. **e**, **f** Western blotting of proteins with/without PX-12 (20 μM, 16 h) treatment. **g** Colony formation assays in HCT-15 cell lines with/without PX-12 (20 μM, 16 h) treatment before irradiation. **h** The survival curves of multi-target single-hit model. **i** Colony formation assays in HCT 116 cell lines with/without PX-12 (20 μM, 16 h) treatment before irradiation. **j** The survival curves of multi-target single-hit model. **k** Overlay of histograms by flow cytometry. **l** Mean of DHE intensity in cells with/without ALDH1L2 knockdown and with/ without PX-12, after 6 Gy irradiation. **m** Apoptosis of different cell lines by flow cytometry. **n** Bar graph to show the percentage of apoptosis cells. **o** Image of tumours from nude mice. **p** Weight of tumours harvested in nude mice. **q** Schematic diagram summarising our working model, namely, decreased ALDH1L2 can interact less with and inhibit the protein degradation of TXN, thereby promoting NF-κB-CAT/SOD2 signaling, followed by increasing ROS scavenge and radioresistance after irradiation. PX-12 inhibits the interaction between ALDH1L2 and TXN and promotes the protein degradation of TXN, and therefore inhibiting the downstream signaling pathway and CRC cells become radiosensitive again. *P < 0.05; **P < 0.01; ***P < 0.001.

We previously found that ALDH1L2 regulates CAT and SOD2 transcription levels [19]. However, to the best of our knowledge, ALDH1L2 has never been reported to be a transcription factor. Based on these data, we hypothesised that there might be an important transcription factor in the mechanism. It is widely known that TXN mediates NF-κB activation. Therefore, we determined the levels of proteins of the NF-κB pathway, which can be regulated by TXN and modulate CAT and SOD2 transcription. In line with our hypothesis, there was an increase in phosphorylated NF-κB protein levels, and a decrease in IκBα levels in ALDH1L2-knockdown cells (Fig. 4c). Contrary results were obtained in ALDH1L2-overexpressing cells (Fig. 4c). In addition, NF-κB shuttled faster from the cytoplasm to the nucleus in ALDH1L2-knockdown cells than in control cells (Fig. 6a, b). Moreover, ALDH1L2 overexpression inhibited NF-κB translocation (Fig. 6c, d). An NF-κB inhibitor, PDTC, was used to assess the impact of NF-κB on downstream gene expression and radiation response (Fig. S6). The results indicated that after inhibition of NF-κB, the protein and mRNA levels of CAT and SOD2 were downregulated, and there was no significant difference when ALDH1L2 was knocked down (Fig. S6A, B). The results of colony formation assay, multi-target single-hit survival analyses and comet assay of the ALDH1L2-NC and ALDH1L2-KD groups did not exhibit any significant differences in the presence of PDTC (Fig. S6C–E).

Next, we used PX-12, a TXN inhibitor, to examine whether it could circumvent the radioresistance induced by the low expression of ALDH1L2. In consonance with the findings of our previous study, first, we found that PX-12 could reverse the upregulation of phosphorylated NF-κB, CAT and SOD2 protein levels in the HCT-15 and HCT 116 cell lines (Fig. 6e, f). Second, the results of colony formation assay and multi-target single-hit survival analyses of the ALDH1L2-NC and ALDH1L2-KD groups did not exhibit any significant differences in the presence of PX-12 (Fig. 6g–j). Next, the results obtained from the analysis of ROS and apoptosis were not different following cell treatment with PX-12 prior to irradiation at 6 Gy (ALDH1L2−, PX-12− versus ALDH1L2−, PX-12+; ALDH1L2+, PX-12− versus ALDH1L2+, PX-12+, Fig. 6k–n). Finally, we verified the role of PX-12 in nude mice (Fig. 6o, p and S7). The results indicated that PX-12 showed significant impact on survival in both HCT-15 cells and HCT 116 cells (HCT-15 NC + DMSO versus HCT-15 NC + PX-12; HCT-15 KD + DMSO versus HCT-15 KD + PX-12; HCT 116 NC + DMSO versus HCT 116 NC + PX-12; HCT 116 KD + DMSO versus HCT 116 KD + PX-12). These also indicated that inhibition of TXN could significantly affect tumour cell survival. Additionally, after PX-12 treatment, there was no significant change between the NC and KD groups (HCT-15 NC + PX-12 versus HCT-15 KD + PX-12). These indicated that ALDH1L2 expression had no effect on survival after TXN inhibition. Of note, tumour weight decreased by ~90% following combined treatment with radiotherapy and PX-12, indicating its possible clinical application (Fig. 6p). In this study, we demonstrated that reduced expression of ALDH1L2 modulates the development of radioresistance in CRC cells by accelerating the TXN/NF-κB signaling pathway and ultimately promoting ROS scavenging. TXN inhibitor reversed the processes and sensitised CRC cells to irradiation (Fig. 6q).

## Discussion

In this study, we elucidated the role of *ALDH1L2* in the radioresistance of CRC and described its functional mechanisms. The key mechanism underlying decreased ALDH1L2-induced enhanced radioresistance can be explained by the fact that due to the downregulation of ALDH1L2 expression, its interaction with TXN is significantly compromised. This inhibits TXN degradation and increases the translocation of NF-κB from the cytoplasm to the nucleus, thereby increasing CAT and SOD2 transcription. Ultimately, this inhibits apoptosis and facilitates survival in excess ROS conditions following irradiation. In the presence of TXN inhibitor, these processes are reversed and thus, PX-12 restricts the development of radioresistance in CRC cells in vitro and in vivo. This study establishes *ALDH1L2* as an effective prognostic marker for CRC radiotherapy and proposes that CRC patients with low levels of ALDH1L2 expression may benefit from the application of TXN inhibitors during radiotherapy.

*ALDH1L2* was discovered relatively recently, and as of now, only a few studies have been conducted on it. It has been reported that ALDH1L2 exhibits abnormal expression levels in several human cancers, including CRC, melanomas, ovarian cancer and pancreatic cancer [9, 15, 17, 37]. Previous studies have shown that ALDH1L2 is upregulated in CRC tissues as compared to normal adjacent tissues [15]. Furthermore, using biological specimens different from those in the TCGA database, recurrence-free survival and overall survival in colorectal patients with high ALDH1L2 enzyme expression were found to be poorer than those in patients with low ALDH1L2 enzyme expression [15]. Of note, a decrease in ALDH1L2 mRNA levels was observed following treatment with oxaliplatin in LoVo cells, a CRC cell line [18]. To the best of our knowledge, no study has yet evaluated the role of ALDH1L2 in CRC radiotherapy.

Through GSEA-based exploration of pathways related to low ALDH1L2 expression, the fatty acid metabolism and oxidative phosphorylation pathways were found to be enriched. A previous study demonstrated that ALDH1L2 dysregulates lipid metabolism in patients with the neuro-ichthyotic syndrome, a rare human disorder [38]. Several studies have shown that ALDH1L2 can generate NADPH, which is an important component of the oxidative phosphorylation pathway [12, 15, 39].

TXN is a classic redox and ROS-related protein that plays a pivotal role in the pathogenesis of CRC. It has been reported that TXN expression is significantly increased in CRC and is associated with the overall reduction in survival [40, 41]. Previous studies have established that enhanced expression of TXN promotes the epithelial-mesenchymal transition of CRC cells and is an independent prognostic marker in patients with CRC having liver metastases [42, 43]. Therefore, TXN is a potential target for drug development to effectively treat CRC. Moreover, TXN inhibitors, such as PX-12 and PMX464, have been used to successfully treat CRC cells. It has been reported that PX-12 causes a rapid decrease (63%) in tumour blood vessel permeability in HT-29 cell-induced xenografts within 2 h of injection [44]. Furthermore, PX-12 not only induces apoptosis but also inhibits migration and invasion of CRC cells [45]. Treatment with PMX464 leads to decreased proliferation and survival of HT-29 cells, and the inhibitory effect of PMX464 is more pronounced under hypoxic conditions [46]. To the best of our knowledge, no study has yet been conducted to evaluate the efficacy of the combined therapy of PX-12 with radiation and their synergistic effect on sensitising CRC cells to radiation. Further, the impact of TXN inhibitors on chemotherapy is largely unknown. Nevertheless, it has been reported that PX-12 restored the sensitivity to cisplatin in human glioblastoma multiforme cells and inhibited the growth of bortezomib-resistant myeloma cells [47, 48].

This study had three main limitations. First, the exact interaction between ALDH1L2 and TXN was not clarified. Although four potential docking models were predicted using H-dock, more experimental evidence is needed. Second, due to the difficulty of obtaining rectal tumour tissues before any treatment, only eight patient tissue samples were used for IHC staining. This may have resulted in a non-negligible offset, and additional samples are needed to draw more accurate conclusions. Third, we focused mainly on how TXN inhibitors affected radiotherapy in tumour cells and did not consider the crucial role of chemotherapy in CRC cancer patients. This limitation will provide direction for our future research.

In summary, based on our data and the fact that once tumours become radioresistant following radiotherapy, patient survival is significantly compromised, it is essential to predict colorectal tumour radioresistance or radiosensitivity before radiotherapy. PX-12, the first TXN inhibitor to undergo clinical development, is a potential therapeutic agent for cancer treatment [49]. As low ALDH1L2 expression results in high TXN expression, PX-12 administration might reverse ALDH1L2-mediated CRC radioresistance. Further investigations are warranted to evaluate and conclusively establish the radiation-sensitising function of TXN inhibitors in physiological and pathological states. This would not only significantly ameliorate the efficacies of the current therapeutic strategies to combat CRC but also help design effective strategies to inhibit the development of radioresistance in cancer cells, thereby improving the clinical outcomes.

## Supplementary information

Supporting information

## Data Availability

Raw mRNA expression profiles and clinical features of the GSE46862, GSE36133, GSE14333 GSE93375 and GSE133057 datasets are available in the GEO database (http://www.ncbi.nlm.nih.gov/geo/). The raw mRNA expression profiles of rectal and colon cancer patients in the TCGA database are available in the National Cancer Institute website (https://portal.gdc.cancer.gov/). We confirm that all the data in this manuscript are original and we have access to the raw data files.
