# Supplementary material for: TXN inhibitor impedes radioresistance of colorectal cancer cells with decreased ALDH1L2 expression via TXN/NF-κB signaling pathway
Source: Br J Cancer. 2022 May 21;127(4):637–48. doi: 10.1038/s41416-022-01835-1 (PMC9381770; doi:10.1038/s41416-022-01835-1)
Supplement: Supplementary file 1 — Supporting information [file 41416_2022_1835_MOESM1_ESM.docx]

**Supporting information**

**TXN Inhibitor Impedes Radioresistance of Colorectal Cancer Cells with Decreased ALDH1L2 Expression via TXN/NF-κB Signaling Pathway**

**Lu Yu et al.**

**Supplementary Figures P2-8**

**Supplementary Tables P9-14**

**Supplementary Figures**

**
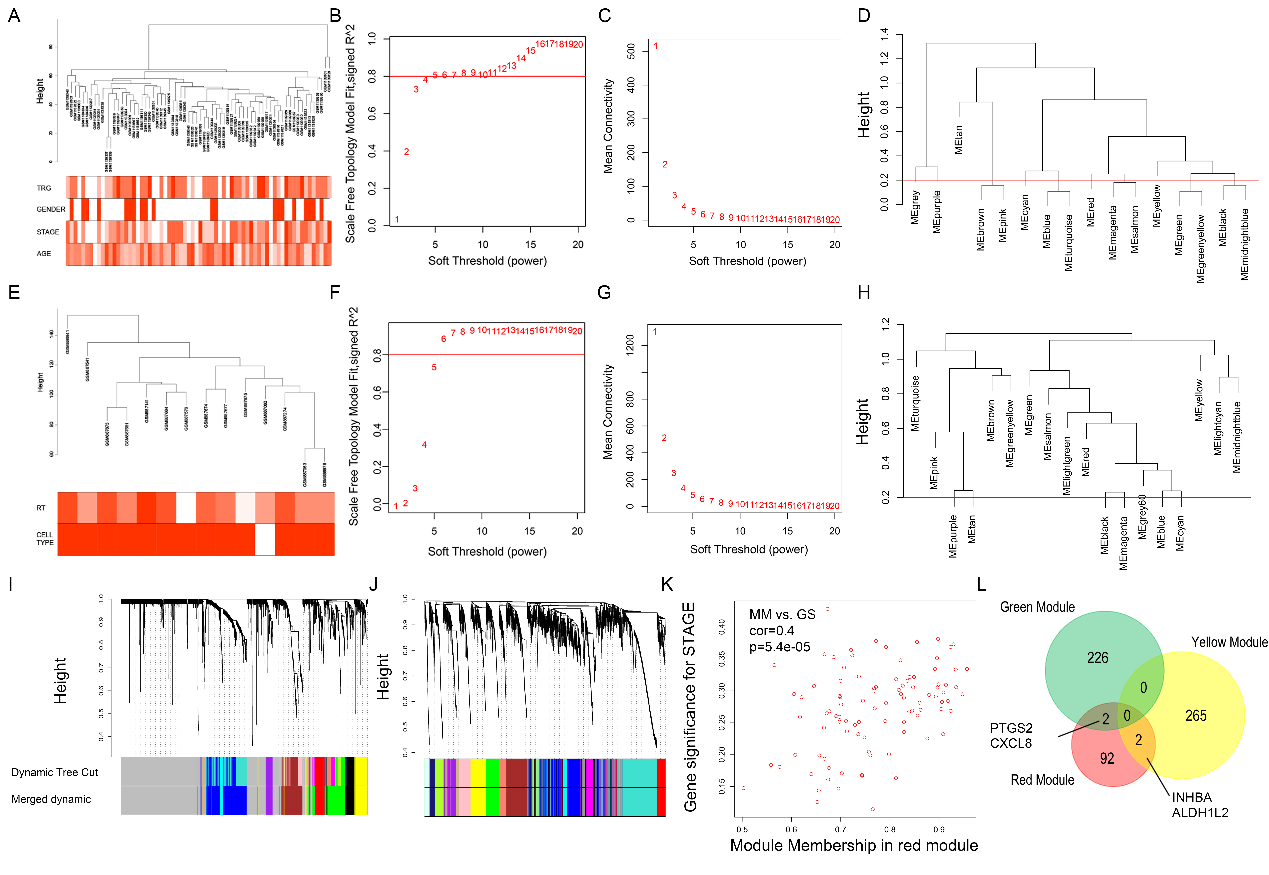
Supplementary Figure 1. WGCNA of GSE46862 and GSE36133 datasets.**

(A) Clustering dendrogram of samples in GSE46862. The clustering was based on the RNA-seq data. Color intensity varies with TRG, gender, stage, and age. In terms of TRG, red means minimal response, and white indicates total response. In terms of GENDER, red means female, and white indicates male. In terms of STAGE, the color changes from white to red, indicating an increase of stage. In terms of age, the redder the color, the older the age. (B) Analysis of the scale-free fit index for various soft-thresholding powers (β = 13). (C) Analysis of the mean connectivity for various soft-thresholding powers. (D) Clustering of module eigengenes in GSE46862. (E) Clustering dendrogram of samples in GSE36133. Color intensity varies with RT and CELL TYPE. In terms of RT, the redder the color, the larger the‾D (Gy). In CELL TYPE, red means colon cancer cell lines and white indicates rectal cell lines. (F) Analysis of the scale-free fit index for various soft-thresholding powers (β = 10). (G) Analysis of the mean connectivity for various soft-thresholding powers. (H) Clustering of module eigengenes in GSE36133. Dendrogram of all genes clustered based on a dissimilarity measure (1-TOM) in (I) GSE46862 and (J) GSE36133 through WGCNA. (K) Scatter plot of module eigengenes in the red module. (L) Veen map of commonly shared genes in red, green, and yellow modules.


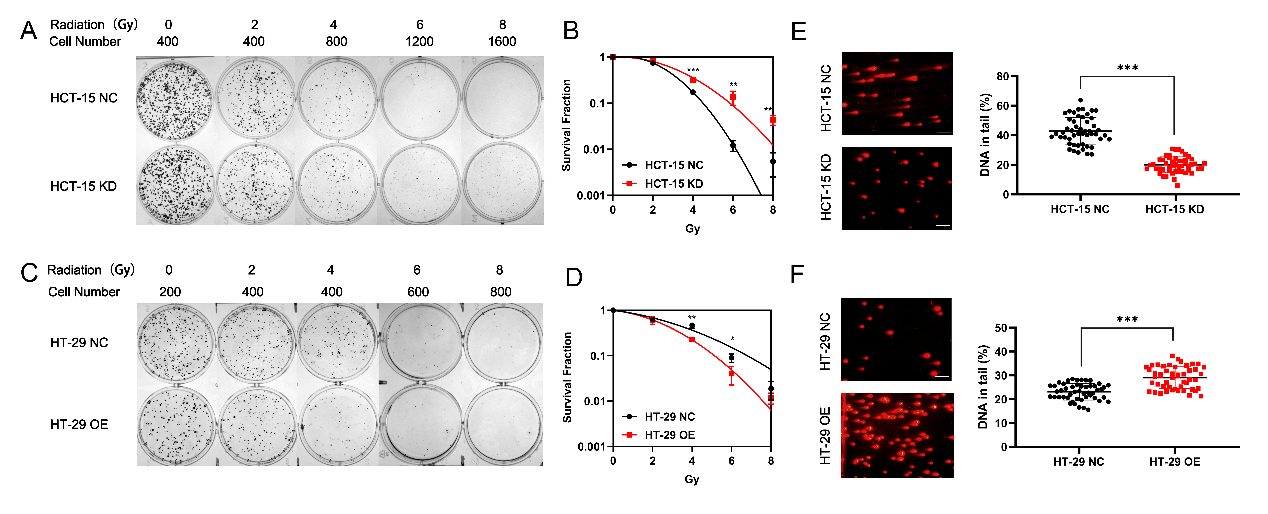


**Supplementary Figure 2. ALDH1L2 is a radiosensitive gene *in vitro*.**

(A) Colony formation assay on ALDH1L2-knockdown HCT-15 cell line and its negative control. (B)The survival curves of multi-target single hit model on HCT-15 cell lines. (C) Colony formation assay on ALDH1L2-overexpressed HT-29 cell line and its negative control. (D)The survival curves of multi-target single hit model on HT-29 cell lines. Comet assay on (E) HCT-15 cell lines and (F) HT-29 cell lines. *P < 0.05; **P < 0.01; ***P < 0.001.

**
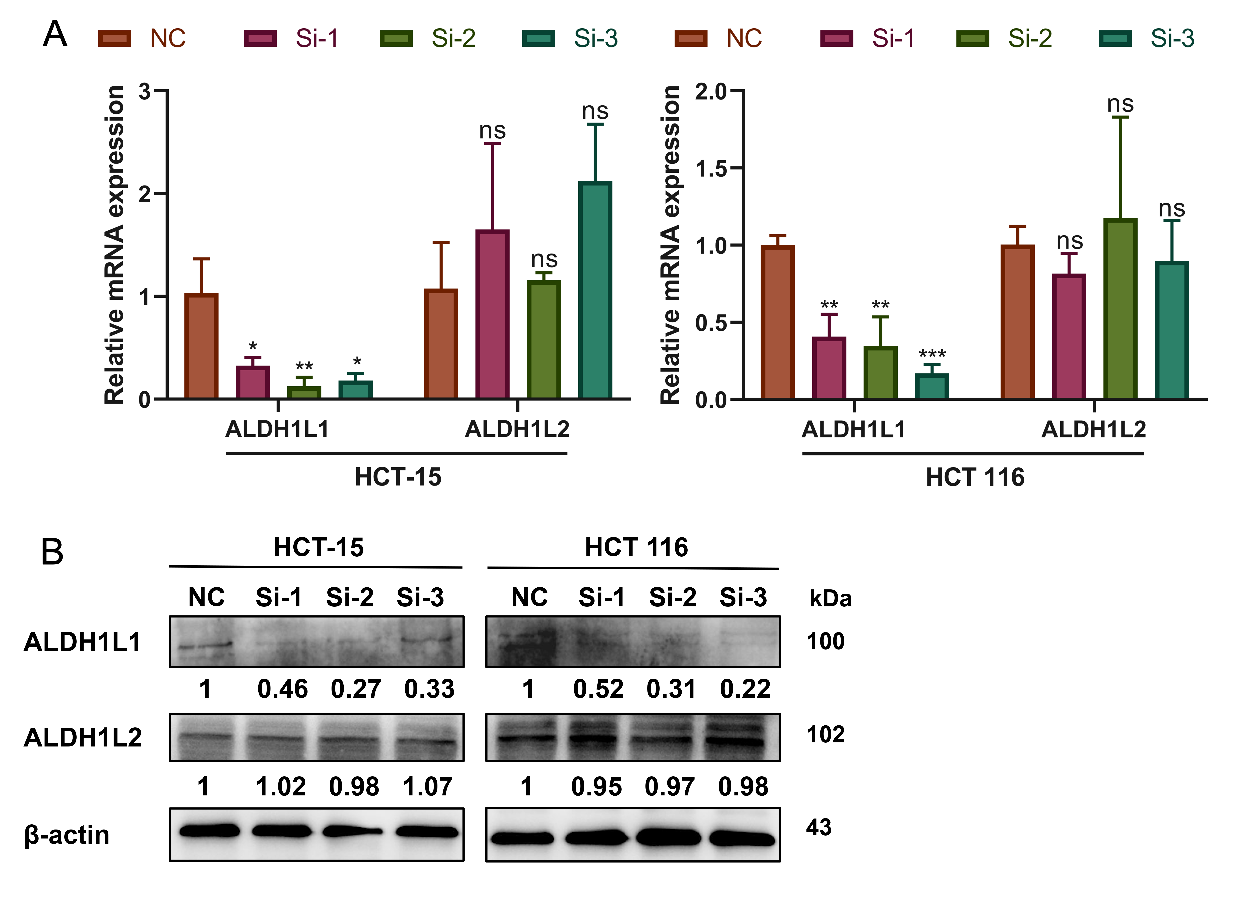
Supplementary Figure 3. ALDH1L2 expression after inhibiting of ALDH1L1.**

(A) ALDH1L1 and ALDH1L2 transcript level in HCT-15 and HCT 116 cell lines after ALDH1L1 was knockdown through siRNA. (B) ALDH1L1 and ALDH1L2 protein level in HCT-15 and HCT 116 cell lines after ALDH1L1 was knockdown through siRNA. *P < 0.05; **P < 0.01; ***P < 0.001.


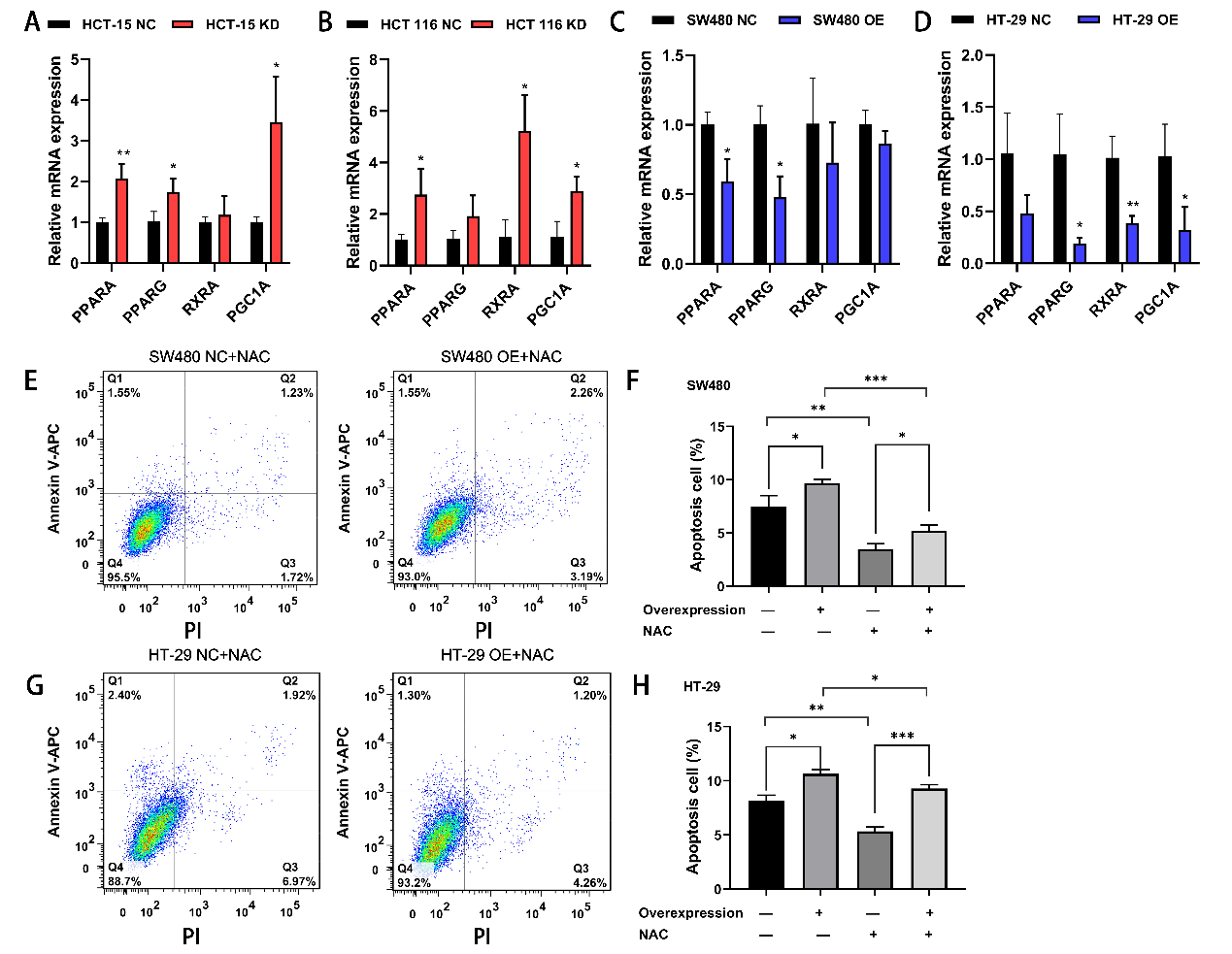
**Supplementary Figure 4. ALDH1L2 regulates fatty acid metabolism and apoptosis.**

(A-D) The mRNA level of genes in the fatty acid metabolism pathway of different cell lines. (E) Apoptosis of different cell lines by flow cytometry of SW480 cell lines, with/ without NAC (5mM, 4h) treatment. (F) Bar graph to show the percentage of apoptosis cells. (G) Apoptosis of different cell lines by flow cytometry of HT-29 cell lines, with/ without NAC (5mM, 4h) treatment. (H) Bar graph to show the percentage of apoptosis cells. *P < 0.05; **P < 0.01; ***P < 0.001.

**
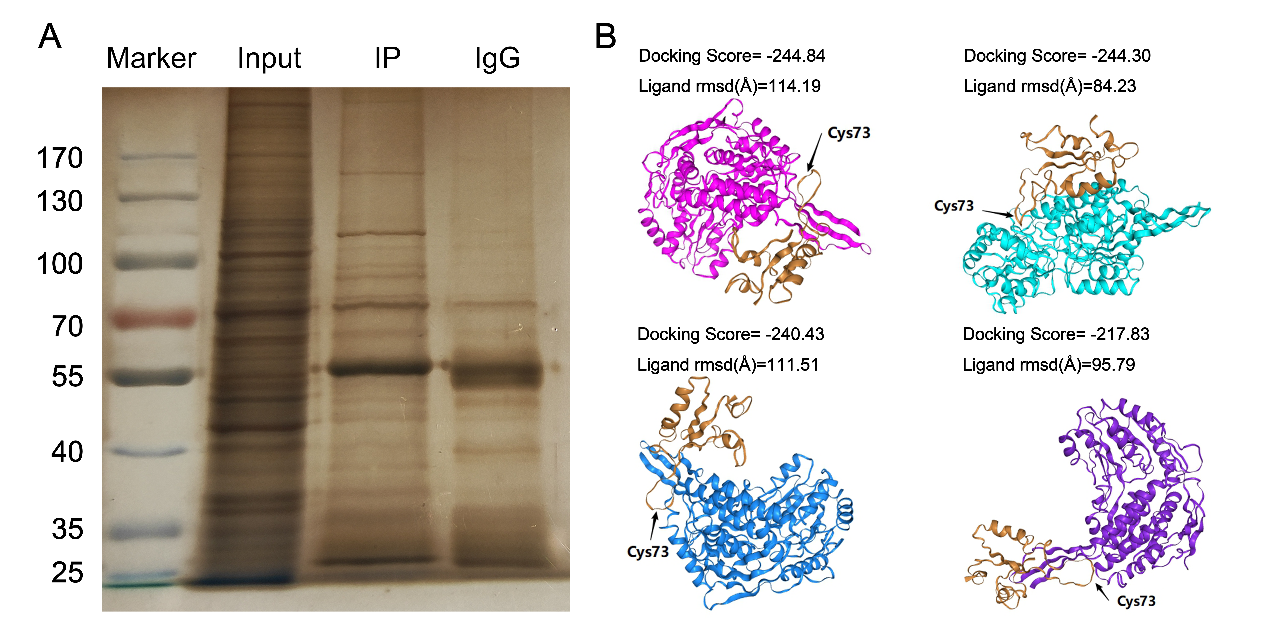
Supplementary Figure 5. TXN interacts with ALDH1L2.**

(A) Image of silver staining. (B) Potential docking model of ALDH1L2 (2O2P) and TXN (4OO4). Cys73 was labeled with the arrow.


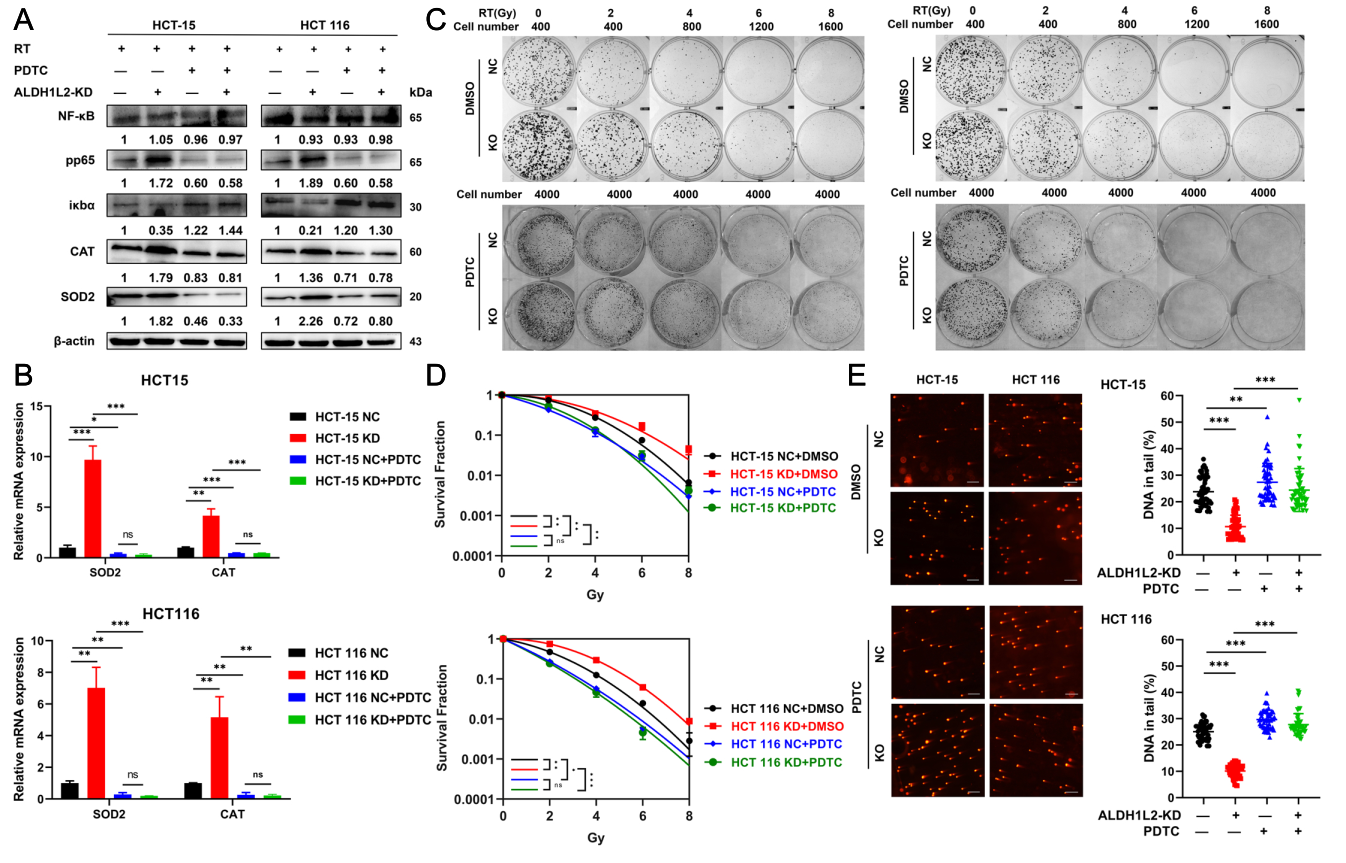


**Supplementary Figure 6. Inhibition of NF-κB after ALDH1L2 was knockdown.**(A) Relative protein level in HCT-15 and HCT 116 cell lines after ALDH1L2 was knockdown and treated with PDTC (100mM, 4h). (B) Relative mRNA level in HCT-15 and HCT 116 cell lines after ALDH1L2 was knockdown and treated with PDTC (100mM, 4h). (C) Colony formation assay on different cell lines and its negative control. (D) The survival curves of multi-target single hit model on HCT-15 and HCT 116 cell lines. (E) Comet assay on different cell lines and its negative control. *P < 0.05; **P < 0.01; ***P < 0.001.


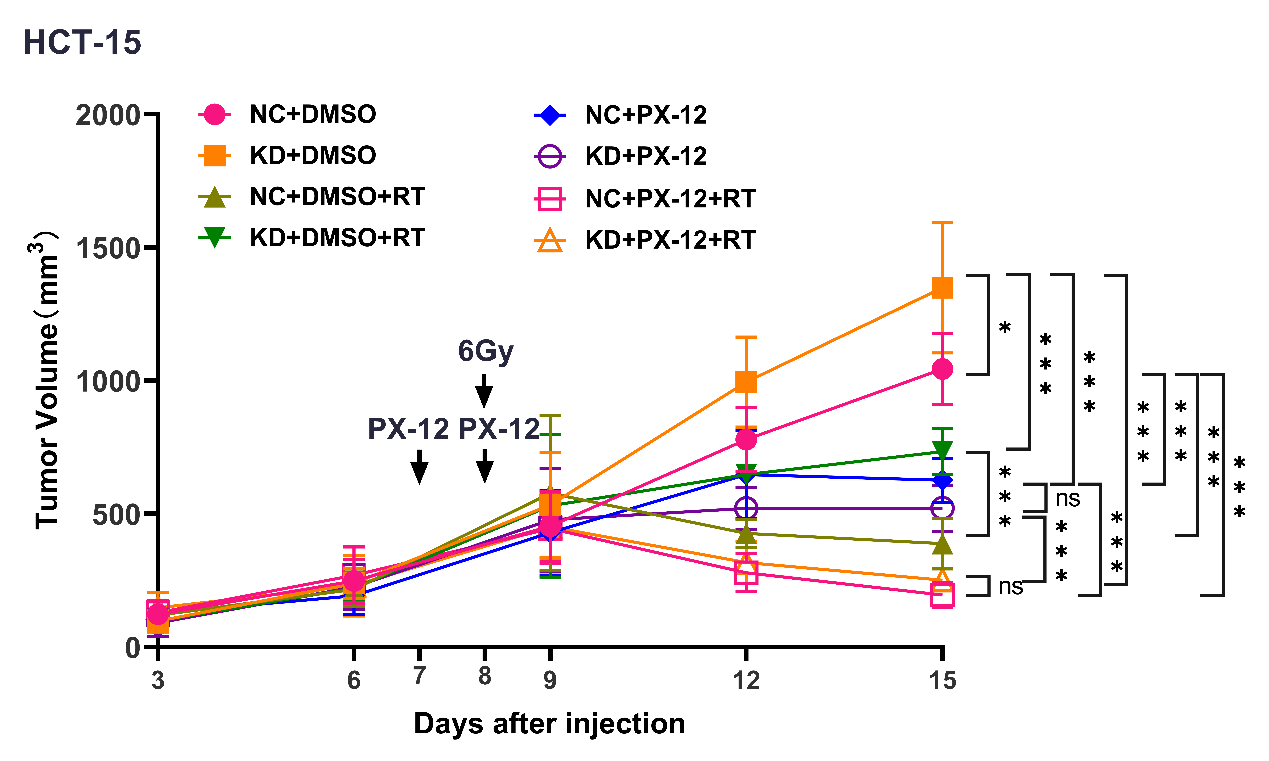
**Supplementary Figure 7. Volume of tumors treated with/without knockdown of ALDH1L2, PX-12 (12mg/kg, i.p.), and 6Gy irradiation.***P < 0.05; **P < 0.01; ***P < 0.001.

**Supplementary Tables**

**Table S1. Reagents**

| Reagent | Company |
| --- | --- |
| Protein extraction kit (KGP113-SDS) | KeyGen BioTech (China) |
| Nuclear and cytoplasmic protein extraction kit  (KGBSP002) | KeyGen BioTech (China) |
| Comet assay kit (KGA240) | KeyGen BioTech (China) |
| Annexin-V APC/PI apoptosis kit (KGA1030) | KeyGen BioTech (China) |
| SYBR Green PCR Kit (RR420Q) | TakaraBio (Japan) |
| cDNA synthesis kit (RR036Q) | TakaraBio (Japan) |
| TRIzol (9108) | TakaraBio (Japan) |
| Pyrrolidinedithiocarbamate ammonium (S3633) | Selleck (USA) |
| Dihydroethidium (S0063) | Beyotime Biotechnology (China) |
| Normal rabbit IgG (A7016) | Beyotime Biotechnology (China) |
| Normal mouse IgG (A7028) | Beyotime Biotechnology (China) |
| DAPI (C1005) | Beyotime Biotechnology (China) |
| Protein A/G PLUS-Agarose (sc-2003) | Santa Cruz (USA) |
| Rabbi anti-ALDH1L2 polyclonal antibody (21391-1-AP) | Proteintech (China) |
| Rabbi anti-ALDH1L1 polyclonal antibody (17390-1-AP) | Proteintech (China) |
| Mouse anti-Flag monoclonal antibody (66008-3-Ig) | Proteintech (China) |
| Rabbit anti-CAT polyclonal antibody (21260-1-AP) | Proteintech (China) |
| Rabbit anti-SOD2 polyclonal antibody (24127-1-AP) | Proteintech (China) |
| Mouse anti-β-actin monoclonal antibody(60008-1-Ig) | Proteintech (China) |
| Mouse anti-Ki67 monoclonal antibody (9449) | Cell Signaling Technology (USA) |
| Rabbit anti-NF-κB p65 monoclonal antibody (8242) | Cell Signaling Technology (USA) |
| Rabbit anti-phospho-NF-κB p65 (Ser536) monoclonal antibody (3033) | Cell Signaling Technology (USA) |
| Anti-mouse IgG, HRP-linked Antibody (7076) | Cell Signaling Technology (USA) |
| Anti-rabbit IgG, HRP-linked Antibody (7074) | Cell Signaling Technology (USA) |
| Rabbit anti-TXN polyclonal antibody (A7638) | Abclonal (China) |
| Goat anti-rabbit IgG/Alexa Fluor 594 (bs-0295G-AF594) | BiossInc (China) |

**Table S2. Primers used for the real-time PCR**

| Gene symbol | Forward Primer | Reverse Primer |
| --- | --- | --- |
| β-actin | CATGTACGTTGCTATCCAGGC | CTCCTTAATGTCACGCACGAT |
| ALDH1L2 | GCTGAAGTTGGCACTAATTGGC | TGAACACCCCTACTACTCGGT |
| ALDH1L1 | AGATTGCAGTGATTGGACAGAG | CCAAAGCCTGGTATTTTGCCA |
| TXN | CTGGATTATGCAGAGTACGTTCG | CGACTTGCTGCTTGCTCAATTT |
| IDH1 | TGTGGTAGAGATGCAAGGAGA | TTGGTGACTTGGTCGTTGGTG |
| IDH2 | CGCCACTATGCCGACAAAAG | ACTGCCAGATAATACGGGTCA |
| HMGCL | ATCTGGACTCCCTGAGTTTGT | GCCAGTTTGGGAAAGTCGATTG |
| CAT | GTCGCCCGTTGTAATAAGGC | TGCTGGCAGTGCTCTTACTTC |
| SOD1 | GGTGGGCCAAAGGATGAAGAG | CCACAAGCCAAACGACTTCC |
| SOD2 | GCTCCGGTTTTGGGGTATCTG | GCGTTGATGTGAGGTTCCAG |
| PRDX5 | GCAAGACGGTGCAGTGAAG | ATGGCATCTCCCACCTTGATT |
| EPHX2 | GTGCTCCGAGACCGCTAAAG | GCTGAAATCGCCTTGTCAAAGAT |
| ACOX1 | ACTCGCAGCCAGCGTTATG | AGGGTCAGCGATGCCAAAC |
| ACAA1 | GCGGTTCTCAAGGACGTGAAT | GTCTCCGGGATGTCACTCAGA |
| AMACR | CCGTTCTGTGCTATGGTCCTG | AGCCTTGGATTTTCCCGCTG |
| ASCL1 | CCCAAGCAAGTCAAGCGACA | AAGCCGCTGAAGTTGAGCC |
| ASCL4 | GGACCAGGCAGAGGAAC | GCAGAGGTGGTAGGCAAA |
| GSTK1 | TCTGGAAAAGATCGCAACGC | GCCCAAAGGCTCCGTATCTG |
| PMVK | CCTTTCGGAAGGACATGATCC | TCTCCGTGTGTCACTCACCA |
| MVK | CCTTTCGGAAGGACATGATCC | TCTCCGTGTGTCACTCACCA |
| RXRA | ATGGACACCAAACATTTCCTGC | GGGAGCTGATGACCGAGAAAG |
| PGC1A | TCTGAGTCTGTATGGAGTGACAT | CCAAGTCGTTCACATCTAGTTCA |
| PPARA | ATGGTGGACACGGAAAGCC | CGATGGATTGCGAAATCTCTTGG |
| PPARG | GATGCCAGCGACTTTGACTC | ACCCACGTCATCTTCAGGGA |

**Table S3. Phenotype data used in WGCNA of GSE46862**

| ID | TRG ^†^ | GENDER ^‡^ | STAGE^§^ | AGE |
| --- | --- | --- | --- | --- |
| GSM1139299 | 2 | 1 | 3 | 68 |
| GSM1139300 | 0 | 1 | 0 | 58 |
| GSM1139301 | 2 | 1 | 1 | 66 |
| GSM1139302 | 0 | 1 | 0 | 56 |
| GSM1139303 | 2 | 2 | 1 | 55 |
| GSM1139304 | 2 | 2 | 2 | 50 |
| GSM1139305 | 2 | 1 | 2 | 37 |
| GSM1139306 | 0 | 2 | 0 | 59 |
| GSM1139307 | 3 | 2 | 3 | 46 |
| GSM1139308 | 3 | 1 | 3 | 68 |
| GSM1139309 | 2 | 2 | 1 | 49 |
| GSM1139310 | 1 | 1 | 3 | 62 |
| GSM1139311 | 2 | 2 | 1 | 65 |
| GSM1139312 | 2 | 1 | 1 | 63 |
| GSM1139313 | 2 | 2 | 2 | 41 |
| GSM1139314 | 0 | 1 | 0 | 33 |
| GSM1139315 | 2 | 1 | 3 | 49 |
| GSM1139316 | 1 | 2 | 1 | 50 |
| GSM1139317 | 2 | 1 | 3 | 73 |
| GSM1139318 | 2 | 1 | 1 | 63 |
| GSM1139319 | 2 | 1 | 2 | 70 |
| GSM1139320 | 2 | 1 | 1 | 69 |
| GSM1139321 | 0 | 2 | 0 | 39 |
| GSM1139322 | 2 | 1 | 3 | 58 |
| GSM1139323 | 1 | 2 | 3 | 41 |
| GSM1139324 | 2 | 1 | 3 | 43 |
| GSM1139325 | 2 | 1 | 3 | 48 |
| GSM1139326 | 1 | 1 | 1 | 72 |
| GSM1139327 | 3 | 1 | 3 | 76 |
| GSM1139328 | 1 | 2 | 1 | 68 |
| GSM1139329 | 1 | 1 | 4 | 62 |
| GSM1139330 | 2 | 2 | 2 | 50 |
| GSM1139331 | 1 | 2 | 2 | 40 |
| GSM1139332 | 0 | 1 | 4 | 62 |
| GSM1139333 | 2 | 2 | 3 | 54 |
| GSM1139334 | 0 | 1 | 0 | 58 |
| GSM1139335 | 2 | 1 | 2 | 45 |
| GSM1139336 | 2 | 1 | 2 | 73 |
| GSM1139337 | 1 | 1 | 1 | 71 |
| GSM1139338 | 0 | 1 | 0 | 52 |
| GSM1139339 | 1 | 1 | 2 | 56 |
| GSM1139340 | 0 | 1 | 0 | 52 |
| GSM1139341 | 0 | 1 | 0 | 39 |
| GSM1139342 | 1 | 1 | 1 | 53 |
| GSM1139343 | 2 | 1 | 1 | 45 |
| GSM1139344 | 2 | 2 | 1 | 67 |
| GSM1139345 | 0 | 1 | 0 | 50 |
| GSM1139346 | 0 | 1 | 0 | 69 |
| GSM1139347 | 3 | 1 | 3 | 61 |
| GSM1139348 | 1 | 1 | 3 | 61 |
| GSM1139349 | 3 | 1 | 1 | 71 |
| GSM1139350 | 3 | 1 | 2 | 62 |
| GSM1139351 | 0 | 2 | 0 | 56 |
| GSM1139352 | 0 | 1 | 0 | 51 |
| GSM1139353 | 2 | 1 | 2 | 50 |
| GSM1139354 | 2 | 2 | 3 | 46 |
| GSM1139355 | 3 | 1 | 2 | 46 |
| GSM1139356 | 2 | 1 | 3 | 44 |
| GSM1139357 | 2 | 2 | 2 | 55 |
| GSM1139358 | 2 | 1 | 3 | 56 |
| GSM1139359 | 3 | 1 | 4 | 73 |
| GSM1139360 | 2 | 1 | 1 | 67 |
| GSM1139361 | 0 | 1 | 0 | 36 |
| GSM1139362 | 3 | 1 | 3 | 60 |
| GSM1139363 | 0 | 1 | 4 | 50 |
| GSM1139364 | 2 | 2 | 3 | 43 |
| GSM1139365 | 2 | 2 | 2 | 41 |
| GSM1139366 | 2 | 1 | 3 | 57 |
| GSM1139367 | 0 | 1 | 0 | 63 |

† TRG means the response of chemoradiotherapy. 0, total response; 1, near total response; 2, moderate response; 3, minimal response.

‡ GENDER means the gender of patients. 1, male; 2, female.

§ STAGE means the tumor stage (UICC-7th). 0, unknown; 1, stage I; 2, stage II; 3, stage III; 4, stage IV.

**Table S4. Phenotype data used in WGCNA of GSE36133**

| ID | RT ^†^ | CELL TYPE ^‡^ |
| --- | --- | --- |
| GSM887674 | 2.36 | 1 |
| GSM887677 | 2.17 | 1 |
| GSM887141 | 2.77 | 1 |
| GSM887063 | 2.02 | 1 |
| GSM886941 | 2.56 | 1 |
| GSM887673 | 2.51 | 1 |
| GSM887681 | 2.08 | 1 |
| GSM887274 | 2.48 | 1 |
| GSM887664 | 2.50 | 1 |
| GSM887675 | 1.20 | 1 |
| GSM887576 | 1.09 | 1 |
| GSM887541 | 1.89 | 1 |
| GSM886979 | 2.02 | 1 |
| GSM887062 | 1.89 | 0 |

† RT means the D (Gy) and the number was according to PMID: 1852919.

‡ CELL TYPE means the type of cancer cell. 1, colon cancer cell line; 0, rectal cancer cell line.

**Table S5. Clinical characteristics of rectal cancer patients**

| Patient | Gender | TRG score^†^ |
| --- | --- | --- |
| Patient1 | Female | 0 |
| Patient2 | Female | 0 |
| Patient3 | Female | 0 |
| Patient4 | Male | 1 |
| Patient5 | Female | 2 |
| Patient6 | Female | 2 |
| Patient7 | Male | 2 |
| Patient8 | Male | 2 |
| Patient9 | Female | 1 |
| Patient10 | Female | 0 |
| Patient11 | Male | 0 |
| Patient12 | Female | 0 |
| Patient13 | Female | 0 |
| Patient14 | Male | 0 |
| Patient15 | Male | 2 |
| Patient16 | Male | 2 |
| Patient17 | Male | 3 |
| Patient18 | Male | 3 |
| Patient19 | Female | 3 |
| Patient20 | Female | 3 |
| Patient21 | Female | 2 |

† The system used to grade tumor response as recommended by the AJCC Cancer Staging Manual, 7th Edition and the CAP Guidelines is that as modified from Ryan R, et al. 0, complete response: No remaining viable cancer cells; 1, moderate response: Only small clusters or single cancer cells remaining; 2, minimal response: Residual cancer remaining, but with predominant fibrosis.
